# Delayed Presentation of Thermal Epiglottitis in a Toddler: A Case Report

**DOI:** 10.7759/cureus.36555

**Published:** 2023-03-22

**Authors:** Nouf Alkaabi, Nouf Aljahdali, Amani Algouhi, Mohammed Asiri

**Affiliations:** 1 Ministry of National Guards Health Affairs (MNGHA), King Abdulaziz Medical City Riyadh, Riyadh, SAU; 2 Pediatric Emergency Medicine, King Abdullah Specialist Children Hospital, Riyadh, SAU; 3 Otolaryngology, King Abdullah Specialist Children Hospital, Riyadh, SAU

**Keywords:** ent - ear nose and throat, pediatrics emergency, airway, burn, thermal epiglottitis

## Abstract

A minor insult to the pediatric airway can have a devastating result. Unfortunately, the signs and symptoms of obstruction might not be present immediately and take some time to develop. Therefore, physicians should have a higher index of suspicion for airway obstruction in children that present with a history of ingestion of scalding liquid. Signs and symptoms of infectious vs noninfectious epiglottis do overlap and the key to differentiate is by careful history and physical exam, especially in nonverbal children. A secondary bacterial infection might complicate thermal epiglottis and make the picture a bit confusing. Therefore, a coordinated approach through a multidisciplinary team is indicated from the start and these cases should be managed and referred to a higher center.

## Introduction

Approaching a child suspected of acute upper airway obstruction needs careful planning. The highest priority is the rapid establishment of a stable and patent airway in a timely manner. Abrupt, precipitous airway obstruction can occur at any moment, hence dealing with such a patient demands extreme caution, and effort should be taken to minimize unnecessary interventions and examinations [1]. Gathering clues from clinical history and physical examination for possible high-risk airways is more important. Calling for help and ensuring the availability of high-risk airway teams is of paramount importance [2]. In this case, we report a two-year-old child who presented with delayed airway obstruction secondary to thermal epiglottitis.

## Case presentation

This is a 27-month-old boy who presented to the emergency department due to difficulty of breathing, attacks of cyanosis, and abnormal breathing sounds. Eight hours prior, the child consulted a primary care physician with a history he spilled hot coffee directly from the thermos onto his face, mouth, and upper chest. There was no history of fever or recent upper respiratory tract symptoms. The child was discharged home at that time.

Upon examination in our emergency department, the child was in a tripod position and having inspiratory stridor with moderate to severe respiratory distress. At the presentation, he was actively crying and maintaining his oxygen saturation on room air. He was kept in his mother's lap without further examination and agitation due to high suspicion of upper airway obstruction. He was noted to have an estimated 2% TBSA burns on chest and face assessed by the palmar method, with no visible oral burn.

The patient was immediately transferred into a resuscitation bay bed in anticipation of airway compromise. Both pediatric otolaryngologist and anesthetist were immediately alerted about the anticipated difficult airway. He started to become lethargic with a weak cry and severe distress. Supportive oxygen 10L via face mask was applied to maintain oxygen saturation above 92%, and he was started on symptomatic management in the form of nebulized epinephrine at 0.25mL and budesonide 2g. Peripheral venous access was obtained, and broad-spectrum antibiotics were started empirically while awaiting transfer to the operating room for a definitive airway. His initial venous blood gas showed ph 7.11 PCo2 72 Po2 41.8 Hco3 22.8 SatO2 57.

**Figure 1:**
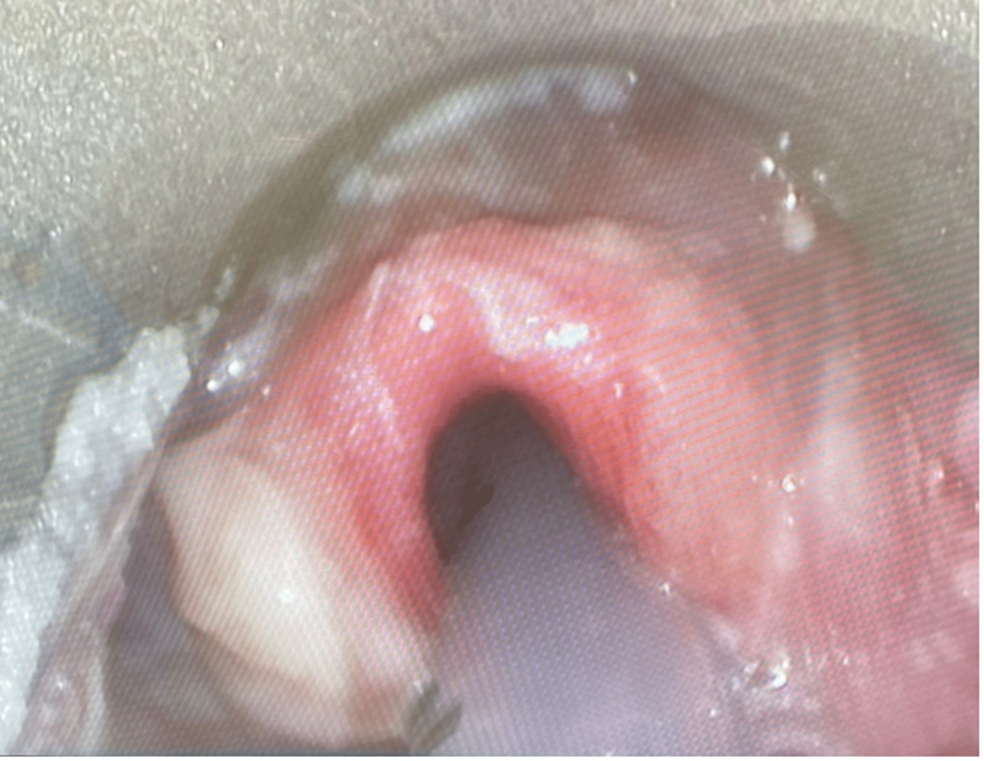
Upper airway examination under general anesthesia revealed severe epiglottic, aryepiglottic fold, arytenoid, and vocal cord edema and erythema.

The patient was transferred from the emergency department to the operating room for direct laryngobronchoscopy and establishing a definitive airway. Multiple intubation attempts by the anesthesia team, using size 3.5 mm and 3 mm endotracheal tubes, were unsuccessful. Using an age-appropriate Parson laryngoscope and utilizing Hopkins rods telescope (0 degrees, 2.9 in diameter, 18 cm in length), examination of the upper airway revealed severe epiglottic, aryepiglottic fold, arytenoid, and vocal cord edema and erythema (Figure 1). However, there was no evidence of necrotic or sloughed tissues. The subglottis and trachea were grossly unremarkable, although full airway assessment was limited due to excessive secretions. With the help of a rigid bronchoscope, intubation was successfully done using an endotracheal tube size of 4 mm. The patient was shifted to the post-operative pediatric intensive care unit for observation.

Due to the thick airway secretions, respiratory cultures and viral studies were collected, and the antibiotics were upgraded after consultation with infectious diseases (ID). Respiratory cultures showed Methicillin sensitive staph aureus (MSSA), and he was managed as concomitant bacterial epiglottitis with appropriate antibiotics based on culture and sensitivity reports.

**Figure 2:**
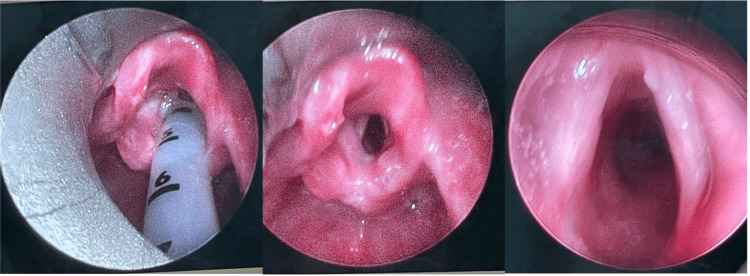
Upper airways examination five days later revealed marked improvement in supraglottic and glottic edema and erythema.

After five days, the patient was scheduled for a second direct laryngobronchoscopy and esophagoscopy. Using age-appropriate Parsons laryngoscope and Hopkins rods telescope (0 degree, 2.9 in diameter, 18 cm in length), examination of upper airways revealed marked improvement in supraglottic and glottic edema and erythema (Figure 2). Patient was extubated, and rigid bronchoscope was further advanced. The subglottic area, trachea, carina, and bilateral main bronchi were normal. No evidence of necrotic or sloughed tissues or foreign bodies was noted. Esophagoscopy revealed intact normal esophageal mucosa. The patient was reintubated with a size 3.5mm endotracheal tube and was transferred back to the pediatric intensive care unit, where he was successfully extubated and then put on a nasal cannula.

## Discussion

Acute upper airway obstruction in pediatrics can be due to many reasons. One of these is epiglottitis, the acute swelling, and inflammation of the epiglottis, which could be due to infectious and noninfectious causes. It is an emergency that requires a high index of suspicion and vigilance to ensure a timely airway intervention.

The primary infectious cause of epiglottitis was historically due to Hemophilus influenza serotype B. Its prevalence was reduced by 99% below the age of five after the introduction of the Hib vaccine in 1985 [3]. Prior to the vaccine, and due to the smaller airway diameter, the condition carried a high mortality rate in children less than five years of age [3]. Noninfectious causes include but are not limited to, local injury following foreign body aspiration and burns, either inhalation or secondary to chemical exposure [4].

In contrast to infectious epiglottitis which can present with fever and leukocytosis, epiglottis secondary to a thermal injury can present with delayed onset of classical signs [5]. Teapot syndrome, which is a thermal epiglottis associated with scalding injury to the face, neck, and thorax, is well described in the literature. It typically occurs when a child grabs hot liquid that tips over and splashes on their face, hands, and upper thorax, leading to facial, peri-oral, and oral burns [6]. The condition can lead to thermal epiglottitis even with the absence of intraoral burns [5]. While it is rare, it is imperative to ensure prompt airway protection [7].

While the condition occurs quickly, it presents itself in different ways. In children, the main symptoms are difficulty breathing and stridor due to the narrowing or obstruction of the airway [6]. Infants often lean forward, sit up, or breathe with their mouths open as they struggle to get more air into their lungs. It is ubiquitous with older children and adults. Additionally, the condition presents itself with difficulty or pain when swallowing.

It should be noted that approximately 20% to 33% of patients with airway burn injuries experience some degree of upper airway obstruction due to pharyngeal edema [8]. Acute airway obstruction is found to be due to supraglottic structure injury [8]. As airway thermal edema is progressive in nature, timely airway management and securing the tracheal airway is the foremost priority in unstable, suspected upper airway burn cases [9]. Airway edema renders nasal/oral intubation challenging [10]. Poiseuille’s law indicates that any reduction in a tubular structure’s diameter will be associated with increased flow resistance to the power of 4. To begin with, pediatric airways are narrow compared to adults; therefore, a minor narrowing can have devastating effects [10].

The increase in resistance in cases of upper airway obstruction, secondary to inhalation injury, can be temporarily reduced by increasing the airway diameter. This can be achieved by using nebulized treatment such as epinephrin (adrenaline) for fast action, and steroids for a delayed but prolonged effect. These interventions can stabilize the child until he can be safely assessed in a proper setting [10].

Management of anticipated difficult airways starts with airway maneuvers and progresses to non-invasive and invasive procedures. Non-invasive procedures include rigid laryngoscopic blades of alternative design and size; using adjuncts (e.g., introducers, bougies, stylets, and alternative tracheal tubes); video laryngoscopes; flexible intubation scopes; supraglottic airway devices; lighted or optical stylets; and rigid bronchoscopes [2].

 In the face of failure to secure the airway with non-invasive procedures, tracheostomy, an invasive procedure, is a known lifesaving alternative [11,12]. In recent studies conducted by Palmieri et al. and Barret et al., it has been observed that tracheostomy is a safe airway rescue procedure in pediatric airway burn patients [13,14]. Another study conducted by Sen et al. examined outcomes of tracheostomy in 45 children who suffered from airway burns among whom 40% had an upper airway burn injury and also proved the safety profile of using tracheostomy in pediatric airway burn cases [15]. Tracheostomy under local anesthesia (awake tracheostomy) might be required in some critical cases [16].

## Conclusions

In conclusion, early recognition and anticipation of impending airway obstruction are of paramount importance in children with airway burns. If faced with this case in a primary care setting, one must anticipate the possibility of airway deterioration and transfer the child to a specialist burn center.

Minimal handling and avoiding unnecessary workup are the way to go to avoid further worsening the already compromised airway. Keeping a broad differential diagnosis of infectious and noninfectious causes helps in prioritizing the management steps until the airway is secured, and so does having a clear difficult airway management pathway. Coordinated team efforts by the emergency physician, the anesthetist, and the otolaryngologist go a long way in the emergency management of these cases.
